# Legume-based green concentrate alleviates negative energy balance and enhances performance, metabolic health, and profitability in postpartum Holstein Friesian cows

**DOI:** 10.14202/vetworld.2025.2414-2426

**Published:** 2025-08-21

**Authors:** Renny Fatmyah Utamy, Ambo Ako, Zyahrul Ramadan, Yasuyuki Ishii, Mohammad Mijanur Rahman, Kannika Umpuch, Azisah Nurfadilah, Gemal Umar Akmal Alkatiry, Muhammad Akram, Jiant Muthahra Maharani

**Affiliations:** 1 Department of Animal Production, Faculty of Animal Science, Hasanuddin University, Jl. Perintis Kemerdekaan KM. 10, Makassar 90245, South Sulawesi, Indonesia; 2 Department of Animal Science, Graduate Student of Animal Science and Technology, Faculty of Animal Science, Hasanuddin University, Makassar 90245, South Sulawesi, Indonesia; 3 Department of Animal and Grassland Sciences, Faculty of Agriculture, University of Miyazaki, Miyazaki 889-2192, Japan; 4 Livestock Production Programme, Faculty of Sustainable Agriculture, Universiti Malaysia Sabah, Sandakan, Sabah, Malaysia; 5 Department of Agriculture, Faculty of Agricultural Technology, Valaya Alongkorn Rajabhat University Under The Royal Patronage, Pathumthani, Thailand; 6 Department of Animal Science, Graduate Student of Animal Science, Faculty of Animal Science, Hasanuddin University, Makassar 90245, South Sulawesi, Indonesia

**Keywords:** beta-hydroxybutyrate, green concentrate, Holstein Friesian, insulin-like growth factor-1, milk production, negative energy balance, sustainable feeding

## Abstract

**Background and Aim:**

Negative energy balance (NEB) is a common metabolic disorder in postpartum dairy cows that compromises milk yield, metabolic health, and reproductive function. Sustainable nutritional interventions are essential to mitigate NEB without increasing production costs. This study evaluated the effect of a legume-based green concentrate (GC) containing *Gliricidia sepium* and *Indigofera zollingeriana* on performance, hormonal profiles, and beta-hydroxybutyrate (BHB) levels in Holstein Friesian (HF) cows experiencing NEB.

**Materials and Methods:**

Eighteen postpartum HF cows diagnosed with NEB (BHB > 1.5 mmol/L) were randomly assigned to one of three treatment groups (n = 6): Commercial concentrate (CON), GC with 20% legume inclusion (GC20), and GC with 30% legume inclusion (GC30). All cows received concentrate at 3% body weight (dry matter basis) alongside elephant grass for 60 days postpartum. Parameters evaluated included milk yield, feed intake, feed conversion efficiency (FCE), body condition score, daily profit, milk composition, and concentrations of BHB, insulin-like growth factor-1 (IGF-1), estrogen, and prolactin.

**Results:**

GC30 significantly improved milk yield (15.88 kg/d), FCE (1.10), and daily profit (United States Dollars 10.99/d), while reducing feed costs and BHB levels in blood (0.91 mmol/L) and milk (0.43 mmol/L) compared to CON (p < 0.05). GC30 also elevated IGF-1 (50.27 ng/mL) and estrogen (104.36 ng/mL), indicating enhanced reproductive readiness. *In vitro* digestibility and rumen fermentation metrics were also superior in GC-supplemented diets, particularly GC30. No adverse effects were observed on prolactin levels or milk protein and lactose content.

**Conclusion:**

Feeding GC, especially GC30, effectively mitigates NEB, enhances productivity, improves hormonal balance, and increases economic returns in postpartum HF cows. This sustainable and cost-effective feeding strategy is suitable for smallholder farmers and supports animal welfare and farm profitability. Future research should explore the effects of long-term GC supplementation and its environmental implications.

## INTRODUCTION

A negative energy balance (NEB) arises when the energy requirements for milk production and phy-siological recovery during early lactation surpass the energy provided by dietary intake. This condition is common in postpartum dairy cows and represents a critical nutritional challenge that, if not properly managed, can compromise lactational performance and overall health throughout the production cycle [1, 2]. NEB is associated with impaired reproductive efficiency, such as delayed uterine involution, and is characterized by the mobilization of body energy reserves – primarily adipose tissue – resulting in a decline in body condition score (BCS). Prolonged nutritional insufficiency can severely affect productivity and animal welfare [3].

During NEB, stored lipids, glycogen, and body proteins are mobilized and metabolized in the liver to meet energy demands. This metabolic process produces beta-hydroxybutyrate (BHB), a key ketone body, whose concentration is positively correlated with the severity of NEB. Elevated BHB levels are indicative of pronounced energy deficits and are commonly associated with hepatic lipidosis and subclinical or clinical ketosis [4, 5]. In addition, NEB can impair mammary gland function, thereby reducing milk synthesis and prolonging the postpartum recovery period [2].

Typically, cows transition out of NEB between 80 and 140 days postpartum, regaining a positive energy balance and stabilizing productivity [6]. The onset, duration, and severity of NEB are influenced by several factors, including prepartum BCS, feed quality, and nutrient digestibility. Nutritional strategies aimed at preventing or alleviating NEB focus on supplying diets rich in protein and energy to meet the heightened metabolic demands of early lactation. Notably, proteins yield approximately 40 adenosine triphosphate (ATP) per molecule, compared to around 36 ATP from glucose, underscoring their substantial contribution to energy supply during this critical phase [7].

Despite the well-documented consequences of NEB in early-lactation dairy cows, including reduced milk yield, impaired reproductive function, and increased metabolic disorders, nutritional strategies targeting its mitigation remain largely dependent on costly com-mercial concentrates (CON). While high-protein, energy-dense feeds are known to support postpartum recovery, the sustainability, affordability, and accessibility of these feeds pose significant challenges, especially for smallholder farmers in developing regions. In recent years, leguminous forages such as *Gliricidia sepium* and *Indigofera zollingeriana* have gained attention for their high crude protein (CP) and fermentable carbohydrate content. However, limited empirical data exist regarding the direct effect of incorporating these legumes into a formulated green concentrate (GC) on NEB-associated biomarkers such as BHB, hormonal indicators (insulin-like growth factor-1 [IGF-1], estrogen, and prolactin), and economic outcomes under field conditions. Most existing studies focus on either *in vitro* digestibility or milk yield, often overlooking the comprehensive physiological, metabolic, and financial responses in cows under NEB stress. Moreover, there is a lack of comparative evidence evaluating varying inclusion lev-els of legume leaves in concentrate formulations, which is essential to optimize feed formulation and guide on-farm applications.

This study aimed to evaluate the efficacy of a legume-based GC,formulated with varying inclusion levels (20% and 30%) of *G. sepium* and *I. zollingeriana* leaves, in mitigating NEB in postpartum Holstein Friesian (HF) cows. Specifically, the study assessed its impact on milk production performance, feed conversion efficiency (FCE), BCS, blood and milk BHB levels, and hormonal profiles, including IGF-1, estrogen, and prolactin. In addition, an economic analysis was conducted to deter-mine the profitability of GC feeding relative to CON supplementation. By integrating nutritional, metabolic, and economic metrics under field conditions, this study provides a comprehensive understanding of how legume-based GC feeding can serve as a sustainable, cost-effective strategy to improve health, productivity, and profitability in dairy farming systems facing the challenge of NEB.

## MATERIALS AND METHODS

### Ethical approval

All experimental procedures were conducted in accordance with the guidelines for institutional animal welfare. The Animal Ethics Committee, Faculty of Veterinary Medicine, Udayana University, approved this study (Approval No. B/184/UN14.2.9/2024).

### Study period and location

The experiment was conducted from August 2024 to February 2025 in Lebang Village, Cendana District, Enrekang Regency, South Sulawesi, Indonesia. This study was conducted under real farm conditions (not a controlled laboratory trial). Therefore, to ensure the accuracy and validity of dairy cattle performance evaluations, sample testing was conducted in a laboratory setting as an indicator for assessing differences among treatments following implementation in dairy cattle under NEB conditions. Hormonal and biochemical analyses were performed at Hasanuddin University Hospital, Makassar, Indonesia. The diet’s chemical composition and *in vitro* digestibility were analyzed at the Dairy Animal Nutrition Laboratory, IPB University, Bogor, West Java.

### Experimental design and animal selection

Eighteen postpartum HF cows (4–5 years old, ~500 kg body weight [BW]) exhibiting signs of NEB were selected for this study. Dairy cattle under NEB conditions were identified through blood BHB analysis. Cows with BHB concentrations exceeding 1.5 mmol/L – indicative of severe NEB – were included in this study. The dairy cattle used in this study were multiparous, with 5–15 lactation days and an average daily milk yield of 11–13 kg. Cows were randomly allocated to three dietary treatments in a completely randomized design (n = 6 per group). The assessed treatments involved different types of concentrates administered to the cows facing the NEB. The treatments included: CON, GC with 20% legume leaves (GC20), and GC with 30% legume leaves (GC30). Randomization was performed using a manual lottery method to ensure equal treat-ment assignment probability for each experimental unit, thereby enhancing the experimental design’s validity and reliability.

### Diet formulation and feeding protocol

**Figure 1:**
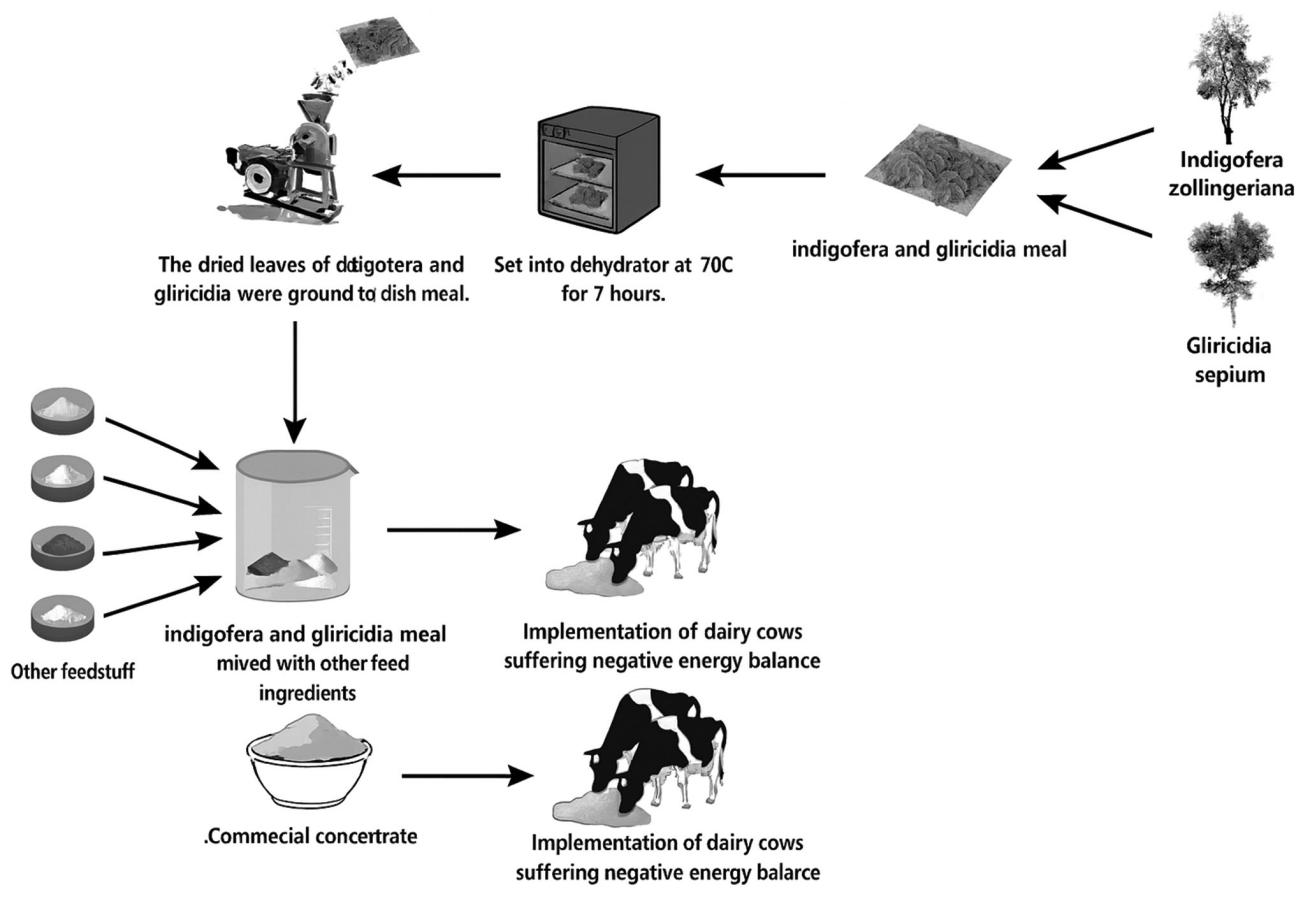
Preparation of green concentrates.

All diets were developed to meet the national standards for lactating dairy cow concentrates (≥16% CP and 70% total digestible nutrient (TDN), as shown in Table 1. Gliricidia and Indigofera leaves were harvested and dried in a dehydrator at 70°C for 7 h. The dried leaves were then ground into a meal using a disk mill (Figure 1). The CON (Rumfeed, CV. Sarana Nutrisi Sembilan, Indonesia). used in this study was sourced from CV Sarana Nutrisi Sembilan, commonly used by local dairy farmers. Cows were fed 3% of their BW (dry matter [DM] basis), with a 30:70 ratio of concentrate to elephant grass (*Pennisetum purpureum*). Concentrate feeding lasted for 60 days postpartum, following a 7-day adaptation period. Cows were housed in individual treatment pens and fed 3 times daily at 06:00, 11:00, and 04:00 a.m.

**Table 1:** Composition of diets under different treatments.

Feedstuffs	Treatments of the diet		

	CON	GC20	GC30
Corn meal	25	20	30
Rice bran	30	23	13
Palm kernel meal (PKM)	10	20	10
Molasses	1	1	1
Fishmeal	17	0	0
Corn cobs	17	0	0
Indigofera leaf meal	0	5	10
Gliricidia leaf meal	0	15	20
Sorghum	0	16	16
Total	100	100	100

CON=Commercial concentrate, GC20=Green concentrate with 20% legume leaves, GC30=Green concentrate with 30% legume leaves

### Sample collection

Milk yield and feed intake were recorded daily throughout the study. Milk and blood samples were collected for laboratory analysis on Day 60. Milk BHB levels were also monitored by collecting samples on Days 0, 30, and 60. Blood samples were collected from one cow per experimental unit using a needle flashback vacutainer, with 3 mL drawn from the jugular vein. Samples were centrifuged at 1,006 × *g* for 10 min to separate serum from blood cells. The resulting serum was transferred into microtubes, stored in a cool box, and transported to the laboratory for analysis using enzyme-linked immunosorbent assay (ELISA). Milk samples for the BHB analysis were obtained from each experimental cow. The samples were initially centri-fuged at 25,155 × *g* for 15 min to separate skim milk from cream. The skim milk was then subjected to a second centrifugation at 2,037,555 × *g* for 30 min to isolate the precipitate. Serum was extracted from the resulting supernatant and stored at −20°C until further analysis.

### Chemical composition analysis of the diet

Feed samples were analyzed for CP (CP; Ass-ociation of Official Analytical Chemists [AOAC] 976.05), crude fat (CF) (CF; AOAC 989.04), crude fiber (CFi) (CFi; AOAC 962.09), nitrogen-free extract (NFE) (NFE; AOAC 2003.05), and ash content (AOAC 942.05). GE was determined using a bomb calorimeter (Model XRY-1A+, China). The calorimeter measured the calorific value based on the heat released during the complete combustion of the sample in excess oxygen [8].

### *In vitro* fermentation and digestibility of rumen

Volatile fatty acids (VFAs) are produced during microbial fermentation of carbohydrates and proteins in the rumen. Major VFAs include acetate, propionate, isopropionate, butyrate, isobutyrate, valerate, and isovalerate. VFA concentrations were analyzed using gas chromatography, based on differential absorption and partitioning across stationary and mobile phases. The separation of VFAs resulted in distinct peaks on the chromatogram. Sample VFA concentrations were quantified by comparing the peak areas to those of known reference standards.

*In vitro* digestibility was assessed following the preparation of McDougall’s buffer and fresh rumen fluid. Each fermenter tube (MAXI-v2, Browin, Poland) was filled with 0.5 g of the sample and 40 mL of McDougall’s buffer. The tubes were incubated in a shaker water bath at 39°C, and 10 mL of rumen fluid was added. A stream of CO_2_ was applied for 30 s, and the pH was adjusted to 6.5–6.9. Tubes were sealed with rubber stoppers and anaerobically incubated for 48 h. After incubation, 2–3 drops of HgCl_2_ were added to each tube to terminate microbial activity. The contents were centrifuged at 2,795 × *g* for 15 min to separate the supernatant and residue. The precipitate was incubated in 0.2% pepsin-HCl solution (50 mL) for 48 h at 39°C. Digested residues were filtered under vacuum using pre-weighed Whatman No. 41 filter paper. The residue-containing filter paper was oven-dried at 105°C for 24 h. The dried samples were weighed to determine the DM content. The samples were ashed at 450°C–600°C for 6 h to calculate the organic matter content. Blank tubes (Fermentation Tubes MAXI-v2, Browin, Poland) (without feed) were included to correct for microbial residue in the CP and GE analyses. Digestibility was calculated using the Tilley and Terry two-stage technique [9] as follows: %IV digestibility = (initial weight [g] − [residual weight (g) − blank correction])/initial weight (g) × 100%.

### Evaluation of animal performance

Milk production and DM intake (DMI) were monit-ored over a 60-day period. FCE was calculated as the ratio of milk yield to DMI (milk yield ÷ DMI). BCS was assessed on Days 0, 15, and 30 of the trial. BCS was used as an indicator of cow health and was determined through visual inspection and palpation. The BCS was evaluated based on eight anatomical landmarks, including the spinous and transverse processes, rumen fill, tuber coxae, tuber ischii, and the area between the hook and tail head. The cows were observed on a flat surface from multiple angles (side, front, and rear) at a distance of 2 m to ensure accurate BCS scoring. The BCS was scored on a scale of 1–5: 1 = emaciated, 2 = thin, 3 = moderate, 4 = fat, and 5 = obese. Intermediate scores (e.g., 0.25, 0.5, and 0.75) were used for more precise BCS estimation.

### Economic analysis

Feed input costs (USD/kg DM) were calculated using the prevailing market prices for each concent-rate component. The GC cost was determined based on the raw ingredients’ current market price. Although forage was home-grown, costs were estimated based on expenses for fertilizer, labor, and transportation. The cost of inputs and outputs included CON at 0.43 USD/kg DM, GC at 0.30 USD/kg DM, grass at 0.15 USD/kg DM, and fresh milk at 0.87 USD/kg.

Daily feed cost (USD/head/day) was calculated by multiplying feed price (USD/kg DM) by feed intake (kg DM/head/day). Daily milk production income (USD/head/day) was calculated as milk yield (kg/day) × milk price (USD/kg). Daily profit (USD/head/day) was obtained by subtracting daily feed cost from daily milk income (profit = milk income − feed cost).

### Assessment of milk quality

Milk quality, including fat, protein, lactose, SNF, inorganic salt, and density, was assessed using an automatic milk analyzer (Infitek MA-H3, China). Milk samples (250 mL) were collected from each cow and transported to the laboratory for analysis. The samples were homogenized and analyzed under controlled laboratory conditions (18°C–24°C). Fifty milliliters of homogenized milk was loaded into the analyzer inlet. The analyzer automatically aspirated the sample and evaluated the milk quality parameters. Results were displayed automatically by the device. Each milk sample was analyzed in duplicate, and the mean of the two measurements was used to interpret the data.

### Hormonal and BHB analysis

#### BHB

Blood BHB concentrations were determined using an ELISA kit (Bovine Beta Hydroxybutyrate, BT Lab, Jiaxing Korain Biotech Co., Ltd, China). Reagents, standards, and samples were prepared according to the kit manufacturer’s protocol. All reagents were equili-brated to room temperature (28°C) before use. Fifty microliters of standard was added to the standard wells; 40 μL of sample and 10 μL of anti-BHB antibody were added to the sample wells. Next, 50 μL of streptavidin-horseradish peroxidase (HRP) was added to each well. Plates were incubated at 37°C for 60 min. The wells were washed 5 times with 300 μL of wash buffer per well. Substrate solutions A and B (50 μL each) were added to each well and incubated for 10 min at 37°C. Then, 50 μL of stop solution was added to each well to terminate the reaction. The optical density was measured at 450 nm using a microplate reader within 10 min of adding the stop solution.

### IGF-1

IGF-1 concentrations were measured using a bovine-specific ELISA kit (Bovine IGF-1, BT Lab, Jiaxing Korain Biotech Co., Ltd.). The procedure started with the preparation of all reagents, standard solutions, and samples, ensuring that all reagents were stored at 28^o^C before use. Fifty microliters of the standard were pipetted into the standard wells; 40 μL of the sample and 10 μL of IGF-1 antibody were added to the sample wells. Streptavidin-HRP (50 μL) was added to all wells, except blanks, and the mixture was gently mixed. Plates were incubated at 37°C for 60 min. After incubation, the sealer was removed, and the wells were washed 5 times with wash buffer, with each well receiving 300 μL of wash buffer and allowed to stand for 1 min. The residual buffer was removed by blotting with absorbent paper. Substrates A and B (50 μL each) were added and incubated for 10 min at 37°C. After this, 50 μL of stop solution was added to each well, leading to a color change from blue to yellow. The absorbance was read at 450 nm using a microplate reader within 10 min of stopping the reaction.

### Estrogen

Serum estrogen levels were quantified using a competitive ELISA kit (Bovine Estrogen, from BT Lab, Jiaxing Korain Biotech Co., Ltd.). All reagents should be acclimated to room temperature (18°C–25°C) before use. A standard curve ranging from 25 pg/mL to 2000 pg/mL was prepared using serial dilutions. The ELISA working procedure is as follows: Begin by adding 25 μL of the standard, sample, and quality control (QC) solutions into each well of the microplate. Next, introduce 200 μL of the HRP estradiol enzyme conjugate (Enzyme Conjugate) into each well and gently shake for approximately 10 s. Plates were incubated at 28^o^C for 90 min. After the incubation period, the solution was discarded from the wells and each well was washed with 300 μL of washing solution. This washing process was repeated 4 times using a microplate strip washer. Once the washing was complete, the wells were gently blotted on absorbent paper to remove excess liquid. Following this, 100 μL of substrate solution (Tetramethylbenzidine [TBM] substrate) was added to each well and incubated for 30 min at 28^o^C. After incubation, the enzymatic reaction was halted by adding 50 μL of stop solution (H_2_SO_4_ 0.5 M). The absorbance was measured at 450 nm using a microplate reader (Bio-Rad, USA).

### Prolactin

Prolactin levels were determined using a com-mercial bovine prolactin ELISA kit (Bovine Prolactin, BT Lab, Jiaxing Korain Biotech Co., Ltd). All reagents and samples were prepared according to the manuf-acturer’s protocol. The necessary number of strips was determined and inserted into the frame. Any unused strips were stored at 2°C–8°C for up to 1 month. For the blank wells, only substrate solutions A, B, and the stop solution were added as controls. Fifty microliters of diluted standard and sample were added to their respective wells. Next, 50 μL of biotin antigen was added to each well, mixed thoroughly, covered with a sealer, and incubated for 60 min at 37°C. After incu-bation, the sealer was removed and the wells were manually washed 5 times with 300 μL of wash solution, blotting after each wash. Then, 50 μL of avidin-HRP conjugate was added and incubated at 37°C for 60 min. After washing, 50 μL each of substrate solutions A and B was added. Plates were incubated for 10 min at 37°C in the dark. The reaction was terminated by adding 50 μL of stop solution. The optical density was recorded at 450 nm using a microplate reader within 10 min.

### Statistical analysis

Data were analyzed using one-way analysis of variance (ANOVA) in the Statistical Package for the Social Sciences version 27.0 (IBM Corp., Armonk, NY, USA). The model used was:

Yij = μ + τi + εij

Where Yij is the observed value, μ is the overall mean, τi is the treatment effect, and εij is the residual error.

Duncan’s multiple range test was used for *post hoc* comparisons where significant differences were found (p < 0.05). Before conducting ANOVA, data normality was assessed using the Shapiro-Wilk test, while Levene’s test was used to evaluate the homogeneity of variances.

## RESULTS

### Chemical composition, rumen fermentation, and digestibility of diets

As shown in Table 2, the GC treatments (GC20 and GC30) exhibited significantly superior chemical composition compared to the CON (p < 0.001). Impro-vements were observed in CP, CF, NFE, ash, CFi, and gross energy (GE) contents.

**Table 2:** Chemical analysis, rumen fermentation, and diet nutrient digestibility.

Parameter	Treatments of the diet			SEM	p-value (values are in two decimals only)

	CON	GC20	GC30		
Chemical analysis (%)					
CP	15.12 ± 0.17^a^	16.12 ± 0.73^b^	17.06 ± 0.24^c^	0.30	0.00
CF	2.90 ± 1.16^a^	6.17 ± 0.20^c^	4.97 ± 0.12^b^	0.47	0.00
CFi	12.96 ± 1.16^b^	4.73 ± 0.11^a^	5.39 ± 0.20^a^	1.33	0.00
NFE	61.57 ± 0.69^a^	66.88 ± 1.11^b^	67.07 ± 0.12^b^	0.92	0.00
Ash	7.44 ± 0.15^b^	5.81 ± 0.33^a^	5.64 ± 0.02^a^	0.29	0.00
GE (kcal/kg)	2668.00 ± 3.00^a^	4078.50 ± 1.50^c^	3981.00 ± 2.00^b^	227.39	0.00
Rumen fermentation (mol/100 mol)					
Acetate	33.61 ± 3.25	29.98 ± 6.47	35.77 ± 5.86	1.76	0.49
Propionate	25.98 ± 2.05^b^	11.18 ± 0.00^a^	28.43 ± 1.54^b^	2.72	0.00
Butirate	6.15 ± 0.36^b^	2.68 ± 0.24^a^	6.63 ± 0.84^b^	0.64	0.00
Iso butirat	0.82 ± 0.05^a^	0.72 ± 0.23^a^	1.73 ± 0.35^b^	0.17	0.00
Valerate	0.82 ± 0.21^b^	0.38 ± 0.06^a^	1.37 ± 0.18^c^	0.17	0.00
Iso valerate	1.14 ± 0.30^b^	0.57 ± 0.06^a^	1.52 ± 0.15^b^	0.14	0.00
VFA (mmol/L)	138.26 ± 2.85^b^	109.24 ± 8.98^a^	134.64 ± 5.03^b^	4.90	0.00
*In vitro* digestibility (%)					
IVDMD	84.12 ± 0.49^a^	87.94 ± 1.10^b^	86.61 ± 0.06^b^	0.59	0.00
IVOMD	83.61 ± 0.52^a^	87.12 ± 0.68^c^	85.59 ± 0.09^b^	0.55	0.00
IVCPD	72.45 ± 0.63^a^	84.97 ± 0.35^c^	83.59 ± 0.70^b^	1.98	0.00
IVGED	71.15 ± 0.89^a^	83.88 ± 0.38^c^	82.23 ± 0.08^b^	2.00	0.00

^a,b,c^Different superscripts in the same row indicate significant differences (p<0.05); SEM=Standard error of the means, CON=Concentrate commercial, GC20=Green concentrate with 20% legume leaves, GC30=Green concentrate with 30% legume leaves, CP=Crude protein, CF=Crude fat, CFi=Crude fiber, NFE=Nitrogen-free extract, IVDMD=*In vitro* dry matter digestibility, IVOMD=*In vitro* organic matter digestibility, IVCPD=*In vitro* crude protein digestibility, IVGED=*In vitro* gross energy digestibility

Rumen fermentation parameters were also signifi-cantly influenced by dietary treatment. GC20 and GC30 significantly increased concentrations of propionate (p < 0.001), butyrate (p < 0.001), isobutyrate (p = 0.005), valerate (p = 0.001), isovalerate (p = 0.003), and total VFAs (p = 0.002). However, there were no significant diff-erences in acetate levels among the groups (p = 0.45).

*In vitro* digestibility metrics were markedly improved in the GC groups. Both GC20 and GC30 showed significantly higher values for *in vitro* DM digestibility (IVDMD) (IVDMD, p = 0.002), *in vitro* organic matter digestibility (IVOMD) (IVOMD, p = 0.004), *in vitro* CP digestibility (IVCPD) (IVCPD, p < 0.001), and *in vitro* GE digestibility (IVGED) (IVGED, p < 0.001) compared to the CON group.

### Productive performance, milk composition, and economic outcomes

As summarized in Table 3, both GC20 and GC30 significantly enhanced milk yield (p = 0.02) and FCE (p = 0.01) in HF dairy cows suffering from NEB. These improvements translated into economic benefits, with GC diets significantly reducing daily feed costs (p < 0.001), and increasing daily milk income (p = 0.03) and daily profit (p = 0.003).

**Table 3:** Performance and metabolic indicators of Holstein Friesian dairy cows fed green concentrates with negative energy balance.

Parameter	Treatments of the diet			SEM	p-value (values are in two decimals only)

	CON	GC20	GC30		
Production performance					
Milk yield (kg/day)	14.37 ± 0.94^a^	15.72 ± 0.49^b^	15.88 ± 0.29^b^	0.25	0.02
DMI (kg/d)	14.34 ± 0.10	14.39 ± 0.13	14.34 ± 0.22	0.04	0.88
FCE	1.00 ± 0.05^a^	1.09 ± 0.02^b^	1.10 ± 0.02^b^	0.01	0.01
BCS	2.62 ± 0.32	2.81 ± 0.12	2.81 ± 0.12	0.06	0.39
Economic benefit					
Feed input (USD/DM kg)					
Concentrate	0.43	0.30	0.30	-	-
Forage	0.15	0.15	0.15	-	-
Daily feed cost (USD/day/head)	3.41 ± 0.01^b^	2.83 ± 0.02^a^	2.82 ± 0.03^a^	0.08	0.00
Daily production income (USD/d/head)	12.56 ± 0.82^a^	13.67 ± 0.25^ab^	13.81 ± 0.43^b^	0.22	0.03
Daily profits (USD/d/head)	9.14 ± 0.80^a^	10.84 ± 0.27^b^	10.99 ± 0.40^b^	0.28	0.00
Milk content					
Fat (%)	3.61 ± 0.20^a^	4.00 ± 0.07^b^	4.28 ± 0.22^b^	0.09	0.00
Protein (%)	3.26 ± 0.05	3.35 ± 0.11	3.37 ± 0.05	0.08	0.16
Lactose (%)	4.96 ± 0.06	4.99 ± 0.14	5.05 ± 0.08	0.02	0.43
SNF (%)	8.90 ± 0.14	9.08 ± 0.24	9.21 ± 0.15	0.06	0.11
Inorganic salt content (%)	0.73 ± 0.01	0.73 ± 0.01	0.74 ± 0.01	0.00	0.49
Density kg/m^3^	1030.79 ± 0.67	1031.85 ± 1.15	1031.48 ± 0.32	0.33	0.46
Kadar BHB dan Hormon					
Blood BHB (mmol/L)	1.19 ± 0.09^b^	0.98 ± 0.12^a^	0.91 ± 0.10^a^	0.04	0.01
BHB milk (mmol/L)	0.63 ± 0.11^b^	0.58 ± 0.09^b^	0.43 ± 0.09^a^	0.03	0.02
IGF-1 (ng/mL)	33.02 ± 3.30^a^	43.36 ± 6.92^ab^	50.27 ± 12.38^b^	3.06	0.05
Estrogen (ng/mL)	76.47 ± 7.99^a^	122.92 ± 5.21^c^	104.36 ± 2.81^b^	5.94	0.00
Prolactin (ng/mL)	66.85 ± 2.53	76.83 ± 13.66	77.97 ± 2.72	2.16	0.16

^a,b,c^Different superscripts in the same row indicate significant differences (p<0.05); SEM=Standard error of the means, CON=Concentrate commercial, GC20=Green concentrate with 20% legume leaves, GC30=Green concentrate with 30% legume leaves, DMI=Dry matter intake, FCE=Feed conversion efficiency, BCS=Body condition score, BHB=Beta-hydroxybutyrate, SNF=Solid non-fat

GC supplementation also resulted in a significant increase in milk fat content (p = 0.002). However, no significant effects were observed on DMI (DMI; p = 0.88), BCS (BCS; p = 0.39), milk protein (p = 0.16), lactose (p = 0.43), solids-not-fat (SNF; p = 0.11), inorganic salts (p = 0.49), or milk density (p = 0.46).

### BHB and hormonal profiles in NEB cows

GC feeding demonstrated a beneficial effect on metabolic and hormonal status in cows experiencing NEB. As shown in Table 3, there was a significant reduction in blood BHB (BHB; p = 0.01) and milk BHB levels (p = 0.02), indicating improved energy balance. In addition, significant increases were observed in estrogen concentrations (p < 0.001) and IGF-1 (IGF-1; p = 0.05), supporting better reproductive and metabolic health.

Conversely, prolactin levels were not significantly affected by dietary treatment (p > 0.05).

## DISCUSSION

### Chemical composition of GCs

The GCs (GC20 and GC30) demonstrated superior chemical profiles compared with the CON (Table 2). GC30 had the highest CP content, indicating that increasing the proportion of gliricidia and indigofera improves dietary protein levels. This aligns with Termizi [10], who reported that legume inclusion, such as indigofera, enhances concentrate protein content, given that both indigofera and gliricidia have a CP content ranging from 27% to 30%. The high CF value observed in GC20 may be due to palm kernel meal, known for its high fiber content (~54%) [11]. The elevated NFE levels in GC20 and GC30 may be attributed to sorghum, a rich source of carbohydrates [12]. Furthermore, the elevated GE levels observed in GC20 and GC30 are a result of the cellulose present in gliricidia and indigofera meal, which serves as an energy source [13]. The increased CFi and ash values are likely due to the inclusion of corn stover and bran in the formulation [14]. Incorporating GC into dairy cow diets can be viewed as an advantageous strategy for those suffering from NEB, given its richness in essential nutrients.

### Rumen fermentation characteristics

The analysis indicated that GC20 resulted in lower total and individual VFA concentrations than GC30 and CON. This indicates suboptimal rumen fermentation in GC20, possibly due to its elevated fat levels (Table 3). Dietary fats are not fermentable and may suppress rumen microbial activity [15]. In fact, high-fat diets (>10%) can inhibit rumen fermentation, potentially reducing microbial efficiency by 50% [16]. The higher levels of VFA observed in the CON diet can be attributed to its greater content of cell wall constituents (CFi), while the elevated cellulose levels in GC30, derived from gliricidia and indigofera, also contributed to the elevated levels of VFA. High CFi diets tend to increase acetate, a precursor for milk fat synthesis. In contrast, diets rich in NFC promote propionate production, contributing to gluconeogenesis in ruminants. Increased propionate levels positively influence energy use [17]. Therefore, the higher energy content in GC30 was anticipated to mitigate, or even eliminate, the occurrence of NEB in postpartum dairy cows.

### *In vitro* digestibility of nutrients

*In vitro* digestibility assessments showed that GC20 and GC30 had higher nutrient digestibility than CON. GC20 showed the highest digestibility, likely due to its lower CFi content. The GC20 treatment showed the lowest CFi content (Table 2), suggesting that higher CFi levels in the feed may decrease its digestibility [18]. This decline not only negatively impacted animal performance but also reduced feed efficiency. Incorporating gliricidia and indigofera into rice straw enhances digestibility [19]. Addition of these high-nutrient ingredients to low-quality feed can significantly improve digestibility. Digestibility is a key indicator of nutrient availability and FE in ruminants. Increased digestibility enhances livestock nutrient intake, making it a vital measure of feed effectiveness, particularly for dairy cows suffering from NEB.

### Milk yield and FCE

**Figure 2:**
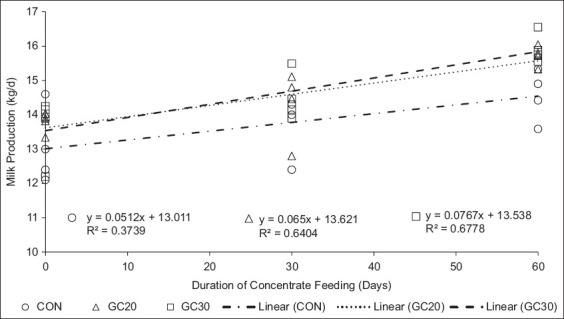
Correlation between milk production and concentrate feeding period An extended duration of concentrate supplementation has shown a positive correlation with milk production and improved nutrient metabolism efficiency. The green concentrate with 20% legume inclusion and green concentrate with 30% legume inclusion formulations, which incorporate legume components and standardized nutritional profiles, significantly enhance milk yield compared with the control treatment.

Cows on GC20 and GC30 produced significantly more milk and had better FCE than those on CON. Milk yield increased from 13.5 kg to 16 kg over 30 days in cows fed GC. This boost in milk output was positively correlated with GC20 (0.64) and GC30 (0.67) levels (Figure 2). Milk yield was influenced by feed quality and diet CP content. Yue *et al*. [20] reported that milk production increased with diets containing 15% CP compared with 13% CP.

Feeding dairy cows with GCs derived from Gliricidia and Indigofera has proven to be an effective strategy for enhancing milk production by fulfilling their nutritional needs. Ako *et al*. [21] revealed that replacing 50% of conventional concentrate with GC can increase milk production and improve milk quality. In addition, the FCE in GC20 and GC30 was superior to the conventional feed. FCE measures how effectively dairy cows convert feed into milk, and feed quality significantly influences its value. In general, higher-quality feed yields better efficiency values [22]. Moreover, FCE is also greatly affected by nutrient digestibility. Arndt *et al*. [23] have shown a marked difference in FCE between dairy cows receiving high-digestibility feed and those consuming low-digestibility alternatives.

### Economic performance and feed affordability

Alongside production traits, economic efficiency of feed is vital for practical application. Economic efficiency can be measured through daily profits, defined as the difference between daily milk production income and feed costs. The CON feed is a commercially available option frequently used by Indonesian dairy farmers; however, its availability is limited, and it tends to be relatively expensive. In contrast, the GC20 and GC30 formulations can be easily adopted by farmers due to the indigenous raw materials near their farms. CON resulted in higher feed costs and lower milk income compared to GC20 and GC30. Daily feed costs are significantly influenced by feed prices and the consumption levels of dairy cows, while milk prices and production volumes determine the income derived from daily production. GC-based feeding strategies effectively reduced input costs and improved milk yield. Using feed technology can lower production costs and allow the incorporation of feed by-products. Ultimately, the goal of minimizing feed costs while optimizing milk production is to improve profitability. Feeding GC20 and GC30 can result in increased daily profits because these feed options are more affordable and capable of boosting milk produc-tion [24]. The adoption of GCs is expected to have a positive impact on farmers’ welfare. This study adopted a strategy focused on using locally available feed resources with optimal nutrient profiles and high digestibility to mitigate the incidence of NEB in dairy cattle. Compared with bypass feeding methods – which may impair rumen function and reduce digestive efficiency – this approach is considered more sustainable. Legume leaves were selected as the primary feed ingredient due to their high nutritional value, cost-effectiveness, and suitability for local cultivation by smallholder farmers, thereby promoting the sustainability of the production system and farmer self-reliance.

### Milk composition and energy balance status

The composition of milk, which encompasses key factors such as milk fat, milk protein, and lactose, serves as a crucial indicator of dairy cow performance. These elements can be used to assess the fresh milk quality [25]. In this study, the quality of milk met the standards established for milk in Indonesia [26]. GC20 and GC30 significantly increased milk fat content, with both CFi content and nutrient digestibility in GC20 and GC30 surpassing those of the control group (CON) (Table 2). Dairy cows fed diets rich in fat sources generally produced higher levels of fatty acids and increased overall milk fat [27]. Higher milk fat levels reflect adequate dietary fat, which reduces the need for body fat mobilization. The absence of fat mobilization suggests that the cows did not experience NEB, which can adversely affect mammary gland performance and, ultimately, milk quality [28].

### BHB and metabolic status

BHB is produced through the catabolism of energy reserves, particularly lipids and glycogen, within the body. BHB levels serve as a primary indicator of the incidence of NEB in dairy cows, with a positive correlation observed between BHB concentrations and the severity of NEB [29]. In addition to its presence in blood, BHB is detected in milk, further correlating with NEB levels. Administration of GCs (GC20 and GC30) has been shown to lower BHB levels in both blood and milk. This reduction can be attributed to the higher GE, NFE, and CP content in GC20 and GC30 compared to the CON diet, thereby providing adequate energy and minimizing lipid and glycogen catabolism, which, in turn, reduces BHB levels. Feeds with elevated GE and NFE levels indicate high carbohydrate content. When consumed by dairy cows, these carbohydrates are converted into glucose, which is subsequently transformed into ATP, a vital energy source for cellular processes – one molecule of glucose produces 36 ATP [30]. Carbohydrates are a primary energy source, but proteins can also provide energy, especially when dairy cows are deficient in blood glucose. Consumed proteins are digested into amino acids, which are then converted into acetyl-CoA and ultimately transformed into ATP [31]. When the energy needs of dairy cows are adequately met, BHB catabolism is reduced. Gibson *et al*. [7] demonstrated that providing high-protein and high-energy diets can improve NEB status in dairy cows.

**Figure 3:**
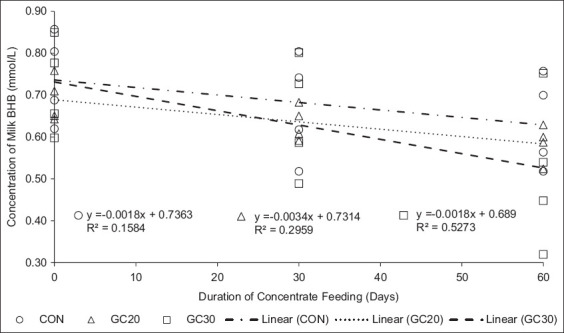
Correlation between milk beta-hydroxybutyrate (BHB) content and concentrate feeding period. Green concentrate with 30% legume inclusion has been shown to significantly reduce milk BHB concentrations.

BHB levels in the blood of dairy cows supplemented with GC20 and GC30 were recorded at 0.98 and 0.91 mmol/L, respectively, indicating a milder state of NEB compared to the CON diet, which exhibited a BHB level of 1.19 mmol/L. According to Chaput and Sirard [32], blood BHB values ranging from 0.59 mmol/L to 0.97 mmol/L suggest mild NEB, while values exceeding 1.0 up to 3.71 mmol/L indicate severe NEB. In terms of milk, a BHB value of ≥0.2 mmol/L signifies that the cows are suffering from NEB [33]. Notably, the lowest milk BHB level observed in the GC30 group was 0.43 mmol/L. Furthermore, GC30 administration significantly reduced BHB levels, demonstrating a strong negative correlation of 0.53 (Figure 3), which underscores its effectiveness in decreasing BHB levels and aiding in the resolution of NEB. NEB typically resolves between 80 and 140 days postpartum, leading to a positive energy balance and improved performance of dairy cows [6]. The duration and impacts of NEB are primarily influenced by factors such as feed digestibility, feed quality, and animal body condition before and after calving.

### Hepatic metabolism and prevention of ketosis

The reduced mobilization of body fat into BHB alleviates hepatic workload. Fat conversion into BHB occurs in the liver and is a metabolically demanding process. If sustained over time, this condition may impair liver function and potentially lead to hepatic lipidosis, commonly referred to as ketosis. In dairy cattle, GC30 has demonstrated the potential to miti-gate the risk of metabolic disorders [34]. GC30 is a sustainable feed innovation that is both safe for animals and economically beneficial for farmers. Furthermore, GC30 has not been previously tested in postpartum cows under NEB conditions. Our study is the first to introduce the use of the GC30 formulation (30% legume meal + 16% CP + 70% TDN), which effectively reduces NEB status.

### IGF-1 response

**Figure 4:**
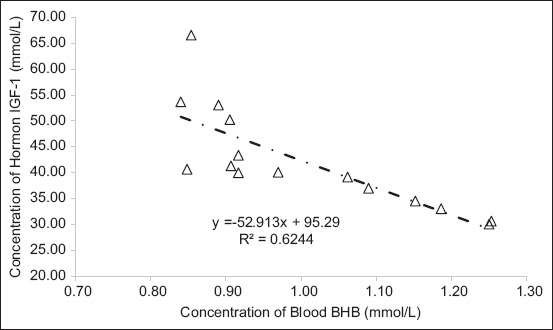
Correlation between blood beta-hydroxybutyrate (BHB) and insulin-like growth factor-1 (IGF-1) the reduction in BHB levels was accompanied by an increase in IGF-1 concentrations.

In addition to BHB, hormone levels such as IGF-1, estrogen, and prolactin are crucial indicators of NEB status. BHB levels were negatively correlated with IGF-1 hormone levels in dairy cows under NEB conditions (Figure 4). A reduction in BHB concentration was asso-ciated with an increase in IGF-1 levels. This inverse relationship is attributed to the elevated BHB production during the acute phase of NEB, where BHB serves as an alternative energy source for the animal. Under such metabolic stress, dairy cattle tend to reallocate nutrient use away from anabolic processes, including hormone synthesis such as IGF-1, to prioritize essential energy demands. Consequently, this shift results in a decline in circulating IGF-1 levels.

IGF-1 plays a key role in restoring normal repr-oductive function during the postpartum recovery period. However, NEB can hinder IGF-1 synthesis, potentially resulting in postpartum anestrus and an extended calving interval. In cows suffering from NEB, IGF-1 levels range from 51 ng/mL ± 8.4 ng/mL in cases of mild NEB to a significantly lower 11 ± 1.1 ng/mL in severe NEB [35]. Nutritional factors, particularly the availability of energy and protein, are essential for regulating IGF-1 production; deficiencies in these nutrients can impede hormone synthesis [36]. Notably, the highest IGF-1 levels recorded in the GC30 treatment were 50.27 ± 12.38 ng/mL, attributed to its superior and more balanced energy and protein content compared to the alternative treatments.

IGF-1 is a peptide hormone comprising a single polypeptide chain consisting of 70 amino acids [37], with leucine being one of the essential amino acids contributing to its structure. GC is particularly rich in amino acids derived from gliricidia (1.38%) and indigofera (2.26%) [38]. The increase in IGF-1 levels observed in the GC30 treatment is supported by the presence of essential amino acids, particularly leucine, which is derived from *G. sepium* at a concentration of 219 mg/g [39]. Leucine serves as a key precursor in IGF-1 biosynthesis [40], thereby contributing to IGF-1 production enhancement in dairy cows receiving the GC30 formulation.

### Estrogen production and CF contribution

In addition to its impact on peptide hormones, NEB limited the synthesis of estrogen hormones. During NEB, fat was utilized as an energy source, resulting in the depletion of precursors essential for estrogen production. Estrogen, a steroid hormone, is synthesized from cholesterol, which is derived from fat. Notably, estrogen levels in the GC20 treatment group were higher than those in the other groups (Table 3). This increase can be attributed to the greater CF content in GC20 than in the other groups (Table 2). The CF in the feed was absorbed and converted into cholesterol in the liver, which acted as an estrogen synthesis precursor [41].

Granulosa cells within ovarian follicles produce estrogen through a series of enzymatic reactions, with cholesterol serving as the primary substrate for its formation [1]. In addition to occurring in GCs, estrogen synthesis occurs in thecal cells. This biosynthetic pathway begins with cholesterol as the primary precursor, which is first converted into pregnenolone, then into progesterone, and subsequently into androgens. The androgens produced in thecal cells are then transported to the granulosa cells to form estrogen [42]. To the best of our knowledge, this is the first report demonstrating a >20% reduction in milk BHB and >50% increase in estrogen following legume-leaf supplementation in NEB cows.

### Prolactin stability and milk production

In addition to its direct effects on milk production, NEB reduces milk yield by restricting prolactin synthesis. Cows with severe NEB typically exhibited prolactin levels below 50 ng/mL [43]. In this study, prolactin hormone levels ranged from 66.85 ng/mL to 77.97 ng/mL. Prolactin, a peptide hormone comprising 199 amino acids, is produced by the anterior pituitary gland and plays a crucial role in milk production [44].

## CONCLUSION

This study demonstrated that dietary suppleme-ntation with GCs containing *G. sepium* and *I. z*ollinge-riana (GC20 and GC30) significantly improved the metabolic, productive, and economic performance of postpartum HF dairy cows experiencing NEB. Both GC formulations, especially GC30, improved feed che-mical composition, enhanced *in vitro* digestibility, and optimized rumen fermentation, as evidenced by significantly higher concentrations of propionate, butyrate, and total VFAs. The GC30 diet showed sup-erior nutritional quality, with higher CP, GE, and NFE content, which contributed to reduced serum and milk BHB concentrations – key biomarkers of NEB – while enhancing energy availability and metabolic balance.

Cows fed GC20 and GC30 produced significantly more milk and exhibited better FCE than those receiving the CON. In addition, the GC diets improved milk fat content and resulted in higher economic returns by reducing daily feed costs and increasing daily profit margins. Hormonal profiles also impro-ved, with significant increases in IGF-1 and estrogen levels, indicating enhanced physiological recovery and reproductive readiness in early postpartum cows. Importantly, this feeding strategy incorporated locally available legume leaves, offering a sustainable, nutritionally rich, and cost-effective alternative to con-ventional commercial diets.

The study’s strengths include the 1^st^-time evalu-ation of the GC30 formulation in postpartum NEB cows, a comprehensive approach combining biochemical, hormonal, production, and economic indicators, and the use of realistic field conditions that reflect actual farm environments. However, the limited 60-day study period and small sample size (n = 18) may restrict generalizability, and long-term impacts on reproductive performance remain unexplored. Future studies should assess reproductive parameters, lactation persistency, and the year-round application of GCs across varied farm systems.

GC supplementation – particularly the GC30 formulation – represents a viable strategy to combat NEB in dairy cows by improving nutrient intake, animal health, milk production, and economic efficiency. Its adoption could enhance smallholder resilience and contribute to sustainable dairy farming systems in tropical regions.

## AUTHORS’ CONTRIBUTIONS

RFU, AA, and ZR: Conceived, designed, and coordinated the study. RFU and ZR: Conducted field sampling, data collection, laboratory work, and data entry. RFU, AA, MMR, and KU: Performed statistical analysis. RFU, ZR, and MMR: Interpreted statistical results. AN, GUAA, MA, and JMM: Conducted field experiments and tabulated the data. All authors contributed to writing, reviewed, and approved the final version of the manuscript.
